# Environmental, Human Health and Socio-Economic Effects of Cement Powders: The Multicriteria Analysis as Decisional Methodology

**DOI:** 10.3390/ijerph14060645

**Published:** 2017-06-16

**Authors:** Laura Moretti, Paola Di Mascio, Simona Bellagamba

**Affiliations:** 1 Department of Civil, Construction and Environmental Engineering, Sapienza University of Rome, 00184 Rome, Italy; paola.dimascio@uniroma1.it; 2 Consulting Engineer, Rome, Italy; simonabellagamba@libero.it

**Keywords:** cement, multicriteria analysis, life cycle assessment, construction industry

## Abstract

The attention to sustainability-related issues has grown fast in recent decades. The experience gained with these themes reveals the importance of considering this topic in the construction industry, which represents an important sector throughout the world. This work consists on conducting a multicriteria analysis of four cement powders, with the objective of calculating and analysing the environmental, human health and socio-economic effects of their production processes. The economic, technical, environmental and safety performances of the examined powders result from official, both internal and public, documents prepared by the producers. The Analytic Hierarchy Process permitted to consider several indicators (i.e., environmental, human health related and socio-economic parameters) and to conduct comprehensive and unbiased analyses which gave the best, most sustainable cement powder. As assumed in this study, the contribution of each considered parameter to the overall sustainability has a different incidence, therefore the procedure could be used to support on-going sustainability efforts under different conditions. The results also prove that it is not appropriate to regard only one parameter to identify the ‘best’ cement powder, but several impact categories should be considered and analysed if there is an interest for pursuing different, often conflicting interests.

## 1. Introduction

The current European Union (EU) regulatory framework concerning the contracting authorities encourages policies inspired by social needs and environmental principles, which contribute to the achievement of sustainable development. Protection and preservation of the natural environment are characterized by three important stages:

1. The transition from development concepts exclusively linked to economic growth, to ones which balance economic, environmental and social issues [1];
2. The definition of environmental protection as an EU constitutional principle [2]: in the last years policies focused on promoting and encouraging the production of more environmentally friendly goods have been adopted;
3. The introduction of the concept of “life cycle” of a product [3,4] and the introduction of “green public procurement” (GPP) [5], applicable to all types of public bids, with the aim of guiding public administrations towards purchasing goods and services less detrimental to the environment [6].

For this purpose, the European Member States have adopted National Action Plans (PANs) [7] to implement GPP procedures in their bids. In Italy, Minimum or Rewarding Environmental Criteria, (MEC or REC), respectively, have been defined: the first ones permit to get into a tender, the second ones allow the competitors to have an additional score. The Italian approach pursues not only environmental but also human health-related objectives: the damage-oriented approach considers damage to human health, ecosystem quality and depletion of resources. This holistic approach is closely related to the growing interest and diffusion of environmental labels which provides guidance on developing criteria, compliance, systems, and operating procedures for awarding sustainability targets [8]. The ISO 14040 series [9,10,11,12,13] sets out three types of labeling scheme: type I is elaborated by a third party, type II is elaborated by the producer, and type III which is based on a full life-cycle assessment, elaborated by the producer and certified by a third party. All three consist of the sustainability performances of the examined product [14], considering its consequences on the environment and human health. However, there are not yet recognized or accredited methodologies to issue such labels: often the approach is subjective and it does not allow a comprehensive analysis. The most frequent adopted methods are based on the interrelation matrices, graphs and check lists, and multi-criteria decision theory [15].

Interrelation matrices permit one to identify and assess the interactions of a project with the surrounding environment. The most known method is the Leopold matrix [16], whose elements represent the environmental impact of each task by means of a couple of values (the coefficients of importance and magnitude). It permits one to compare different alternatives, identifying the projects with maximum positive or negative impact. The procedure does not consider the interaction between effects, and does not give criteria to define the importance and magnitude coefficients, therefore it does not ensure reliable results. Graphs (or networks) derive from interrelation matrices, but they do not allow quantitative and comparative analyses. Check lists consist of qualitative environmental, social and economic elements or quantitative reference lists to be considered in the calculation of impacts. The Environmental Evaluation System, known also as the Battelle method [17,18], is the best known. This method does not consider existing uncertainties between objective and subjective values of indicators and their functions. Moreover, it does not consider the possibility of interaction with the community [19] for defining parameters, especially in case of human and social factors. The models based on the “multi-criteria decision” theory permit one to identify the best alternative, to get a ranking of alternatives, to reduce the number of alternatives to be analyzed, and to distinguish between acceptable and not acceptable alternatives. In this context, the Analytic Hierarchy Process (AHP) is particularly suitable when most costs and benefits are intangible, and it is not possible to compare economic data.

This last methodology has been used in the paper, which compares the environmental, human health and social effects [20] of cement, the most used material on the world [21] for structure and infrastructure construction [22,23,24,25,26]. The study considers four powders whose “environmental” performances have been certified by mean Environmental Product Declarations (EPD), which are type III labels [27]. Workers’ safety and health, and economic data derive from internal documents produced by the companies. The results permit to compare the sustainability of the cements, and identifying the strengths and weaknesses of the examined powders.

The methodology herein adopted could be efficiently used and modified to analyze other products or services: it is a versatile and unbiased tool to achieve the sustainable policies encouraged by the European Community legislature.

## 2. Materials and Methods

The authors considered four gray cements prepared by four different Italian companies, which are not named for reasons of confidentiality. All cements guarantee 32.5 MPa of 28-day characteristic cubic compressive resistance (*R_ck_*). All powders are Portland-pozzolana cements, defined as CEM II according to [28]: they have 65–89% of clinker, 21–35% of natural pozzolana, and 0–5% of other constituents. Although each clinker content is not known by the authors, however the powders are comparable because they represent specific, detailed and homogeneous chemical and mechanical characteristics. As regard to as the environmental and social criteria, the study considers the impact categories listed in Table 1: their values refer to the production of 1 Mg of cement. According to the standard EN 15804 [29], these categories describe environmental impacts, resource use, and waste categories related to a production. They are therefore representative of environmental and human health consequences of a manufacturing process.

As regard to the human health protection criteria, the authors considered the most common risks for construction industry workers [30]: noise, whole body vibrations (WBV), hand-arm vibrations (HAV), exposure to allergizing substances, exposure to Cr(VI) (hexavalent chromium), and exposure to free crystalline silica (FCS). All values are deduced from the (internal) Document of Risk Evaluation prepared by each company, as required by the Italian standard about the safety of workers [31]. As regard as the content of Cr(VI), the Italian companies comply with the European Directive 2003/53/CE [32] and doubly test their cements to increase worker safety [33]. As regard to the exposure to FCS, the weekly Permissible Exposure Limit considered in the study is compliant with the American Conference of Governmental Industrial Hygienists standards [34]. Examined risks have been classified according to the standardization proposed by the Italian Ministry of Labour [35].

For the economic issues, the authors considered the unit price of cements, listed on the pricelist of each cement plant (not herein disclosed due to privacy reasons). Table 2 lists the human health and economic data.

The impact categories listed in Table 1 and Table 2 have been considered as decision criteria to choose the most sustainable cement powder by mean the AHP method. It consists of decomposition of decision problem (what is the best, or most sustainable, cement?) into hierarchy of easier sub-problems, each independent from others. Each element of the hierarchy could be referred to any aspect of the decision problem (e.g., both tangible and intangible, measured and estimated variables).

**Figure 1:**
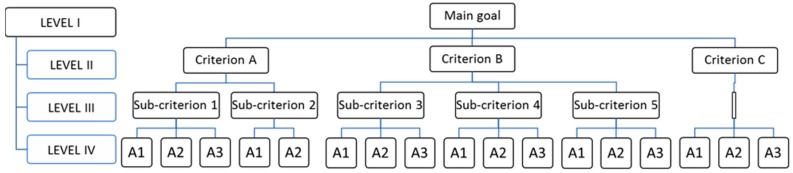
Example of hierarchical decomposition.

The method is composed of three phases:

1. Hierarchical decomposition, which includes:
  - a first level, which represents the overall “goal” of the analysis;
  - a final level, which represents the alternatives to be considered;
  - intermediary levels, which represent criteria and subcriteria for evaluating the alternatives (named A1, A2, A3), as represented in Figure 1.

Figure 1 represents four levels of analysis. The hierarchical decomposition consists of three criteria (criterion A, B and C) and five sub-criteria (sub-criterion 1 to 5) to be applied to three alternatives.

2. Pairwise comparison: each element of each level is compared to other elements of its level respect to each criterion of the higher level. The decision maker should answer questions such as: “how much is the alternative i preferable with respect to the alternative j from the point of view of the k criterion?” or “how much is the criterion l most important than the criterion m with respect to the general objective?” The answer should be quantitative, therefore when it is not possible the relative scale of importance by Saaty [36] could be used. This method determines the relative importance of criteria by mean the pairwise comparison, using the numerical/semantic scale represented in Table 3.

The scale of importance shown in Table 3 allows the decider composing the matrices pairwise comparisons (*C*), whose *e_ij_* elements means the preference of *i* with respect to *j* and the *e_ji_* are reciprocal of *e_ij_* (Table 4). As consequence of this, such matrices are square and have dimensions equal to the number of variables (*O_i_*) considered elements in the hierarchical level under consideration.

For each hierarchical level, the decision maker generates as many pairwise comparison matrices as elements of the upper level. From each of these matrices, elements of the considered hierarchical level are ordered respect to each involved criterion of the upper level. A pairwise comparison matrix is consistent if any three elements *e_ij_*, *e_ik_*, *e_jk_* satisfies Equation (1) [37]:

*e_ik_ = e_ij_ e_jk_* (1)

3. Hierarchical re-composition: from the vector of preferences of each lower level respect to its upper level, it is possible to obtain the vector of overall ordering vector of alternatives in relation to the goal. In presence of 3-level hierarchy, as represented in Figure 1, ordering vectors are those of alternatives compared to each criterion and those of criteria compared to the goal.

Compared to benefit/cost analyses, the evaluation in the AHP process does not seek to find the “optimum project”, but to find a satisfactory solution to several, often intangible, objectives considered in the decisional process. According to the Pareto efficiency, the analysis is based on optimum allocation of available resources [38]. The hierarchical analysis is mathematically more complex than classic multi-criteria analysis [39], but it simplifies the work of decision maker, which responds to simple and same type questions, the pairwise comparisons. Subjective elements, as the choice of the relative importance scale, and the definition of the acceptable threshold inconsistency, are present in the hierarchical analysis and they limit the action of decision maker, but the method ensures an inclusive approach to multi-criteria problems. Indeed, it allows the evaluation of multiple aspects, as happens in construction bids, where legislative, technical, economic and environmental issues should be considered. On other hand, the method is decried as being arbitrary in the assignment of the relative weights and influenced in the result by the number of considered alternatives. As regard as the first weak point, the authors interviewed technicians from different backgrounds, experts in the fields of environment, human health and economy (i.e., data listed in Table 1 and Table 2). As regard as the latter weak point, the analysis considers only ten impact categories instead of 26 defined by [29], and limits human health and economic analysis to the most considered variables at international level [40].

## 3. Results

**Figure 2:**
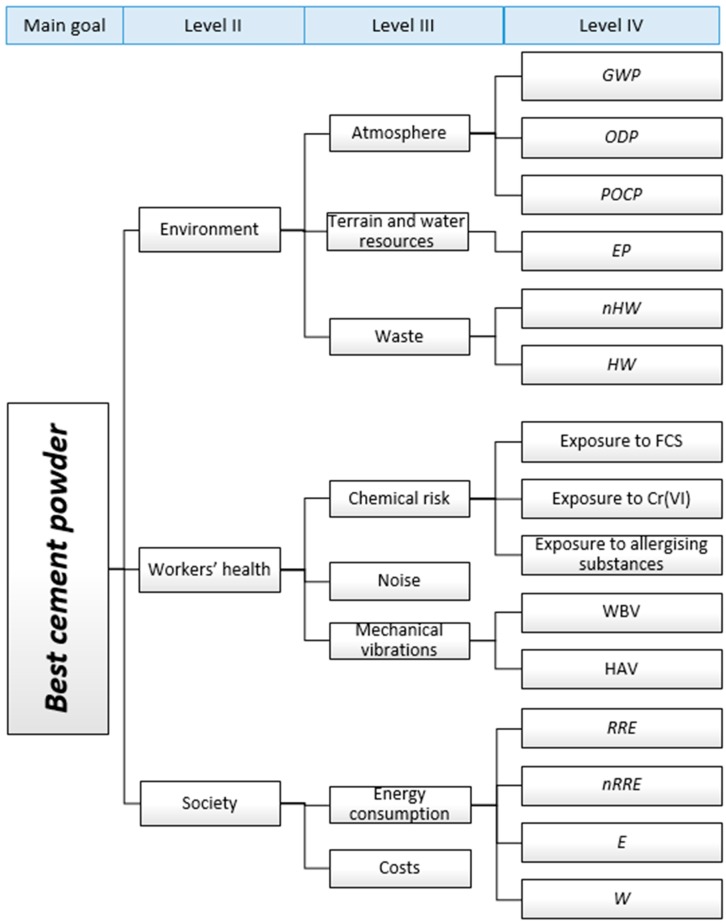
Hierarchical decomposition used for the analysis. *GWP*: global warming potential; *ODP*: ozone depletion potential; *POPC*: photochemical ozone creation potential; *EP*: Eutrophication potential; *nHW*: non-hazardous waste; *HW*: hazardous waste; FCS: free crystalline silica; WBV: whole body vibrations; HAV: hand-arm vibrations; *RRE*: renewable resources with energy content; *nRRE*: non-renewable resources with energy content; *E*: electricity; *W*: water consumption.

The study focused on the choice of the most sustainable cement powder to be used for cement bound mixtures (e.g., grouts, mortars, concrete). The four cement powders are identified by the nomenclature *cement n*, with *n* varying from 1 to 4, and they are produced by different plants numbered according to the cement nomenclature (i.e., cement 1 is produced by plant 1 or company 1). The hierarchy defined by the authors involves four level of analysis, as represented in Figure 2:

(1) The main goal, the choice of the best cement powder (level I);
(2) The objectives of (level II):
  - Minimizing environmental interferences,
  - Minimizing negative social-economic aspects,
  - Maximizing health of workers reducing their job risk;
(3) The objectives of (level III):
  - Minimizing impacts on the atmosphere, terrain and water resources (sub-objectives of environment analysis),
  - Minimizing the effects of noise, chemical substances and mechanical vibrations (sub-objectives of workers’ health analysis),
  - Minimizing the energy consumption and costs (sub-objectives of socio-economic analysis);
(4) The objectives of (level IV):
  - Minimizing global warming potential *(GWP)*, ozone depletion potential *(ODP)*, and photochemical ozone creation potential *(POCP)* (sub-objectives of atmosphere analysis),
  - Minimizing eutrophication potential *(EP)* (sub-objective of water environment analysis),
  - Minimizing non-hazardous and hazardous waste (sub-objectives of terrain analysis),
  - Minimizing exposure to free crystalline silica, Chromium VI and allergizing substances (sub-objectives of chemical risk analysis for workers),
  - Minimizing exposure to whole body vibrations and hand-arm vibrations (sub-objectives of mechanical risk analysis for workers),
  - Minimizing consumption of renewable resources with energy content, non-renewable resources with energy content, electricity, and water.

As regard as the priorities relating to the three criteria of level II, pairwise comparisons gave the results shown in Table 5. Thirty two (32) technicians have participated in the AHP analysis: eight environmental engineers, eight chemical engineers, eight occupational physicians, and eight energy managers. The geometric mean has been used to aggregate individual judgements and obtain pairwise comparison matrices. Values of *c_ij_* show little preference for workers’ health respect to environment and society, consistently with the main goal of “sustainability”.

Each element *c_ij_* derives from application of the Saaty method [36]: it represents the importance relationships between each pair of criteria.

Pairwise comparison matrix for level II (C,_II_) has been normalized. Each element *x_ij_* of the normalized pairwise comparison matrix for level II (N,_II_) is obtained by dividing each element *c_ij_* by the sum of each column of pairwise comparison matrix (Equation (2)):

(2) $$x_{ij}=\frac{C_{ij}}{\sum_{j=1}^{n}C_{ij}}$$

Table 6 represents the normalized pairwise comparison matrix (N,_II_) for level II.

Table 6 allowed the calculation of weights to be related to each criterion of level II. Weights *w_ij_* have been obtained by Equation (3):

(3) $$w_{ij}=\;\frac{\sum_{j=1}^{m}X_{ij}}{m}$$

Weight vector of level II (W,_II_) is represented in Table 7.

The analysis of consistency was composed of three phases:

- Analysis of consistency of each criterion and assessment of consistency vector;
- Measure of consistency by mean consistency index (*CI*) as deviation or degree of consistency according to Equation (4):
  (4) $$CI=\;\frac{l_{max}-n}{n-1}$$
  where *n* is the size of the pairwise comparison matrix *C* and *l_max_* is the largest eigenvalue of *C* when *w* is the weight vector, *l_max_* satisfies Equation (5):
  (5) $$C\cdot w=l_{max}\cdot w$$
- Measure of the ratio consistency (RC) according to Equation (6):
  (6) $$RC=\;\frac{CI}{RCI}$$
  where *RCI* is the Random Consistency Index proposed by Saaty for different size of the comparison matrix (Table 8).

It is acceptable *RC* ≤ 1; other values require revision of the subjective judgements.

The analysis of consistency for level II gave the results listed in Table 9 (*l_max_* is 3.05).

The results listed in Table 9 satisfy the condition proposed by Saaty [36]: the judgements are within the limit of consistency. As regard as the priorities relating to criteria of level III, pairwise comparisons for environment analysis gave the results shown in Table 10.

Table 11 shows the normalized pairwise comparison matrix for level III, environment analysis (N,_IIIe_).

Weight vector of level III, environment analysis (W,_IIIe_) is represented in Table 12.

The analysis of consistency of level III, environment analysis gave the results listed in Table 13: they satisfy the condition proposed by Saaty [36] (*l_max_* is 3).

Similar procedure has been applied to criteria “worker’s health” and “socio-economical” aspects. For the first one the results are based on the pairwise comparison matrix for level II, shown below in Table 14.

Weight vector for level III, workers’ health analysis is represented in Table 15.

As regard as the priorities relating to criteria of level IV, pairwise comparisons for atmosphere analysis gave the results shown in Table 16.

Table 17 shows the normalized pairwise comparison matrix for level IV, atmosphere analysis (N,_IVa_).

Weight vector of level IV, atmosphere analysis (W,_IVa_) is represented in Table 18.

The analysis of consistency of level IV, atmosphere analysis gave the results listed in Table 19: they satisfy the condition proposed by Saaty [36] (*l_max_* is 3.11).

Similar procedure has been applied to sub-criteria waste, water environment, energy.

At the end of the hierarchical analysis involving all sub-criteria, the cements have been compared. Table 20 shows the comparison matrix for environmental sub-criteria *GWP*.

Table 21 shows the normalized pairwise comparison matrix for environmental sub-criteria *GWP*.

Weight vector of *GWP* for the examined alternatives is represented in Table 22.

The analysis of consistency of environmental sub-criterion *GWP* gave the results listed in Table 23: they satisfy the condition proposed by Saaty [36] (*l_max_* is 4.15).

Similar procedure has been applied to all considered sub-criteria of level IV. Considering the level IV of the criterion “atmosphere”, the corresponding sorting matrix of alternatives with respect to considered sub-criteria is composed of the vertical weight vectors calculated for each sub-criterion considered in the level under criterion *atmosphere* (Table 24).

Performances of cements respect to atmosphere are calculated by mean the hierarchical re-composition of the analysis. In particular, the sorting matrix of alternatives, atmosphere shown in Table 24 should be multiplied by:

- The sorting vertical vector calculated for level IV, *atmosphere* (Table 18);
- The weight of atmosphere in the vertical weight vector for level III (Table 15);
- The weight of environment in the vertical weight vector for level II (W_,II_) (Table 7).

**Figure 3:**
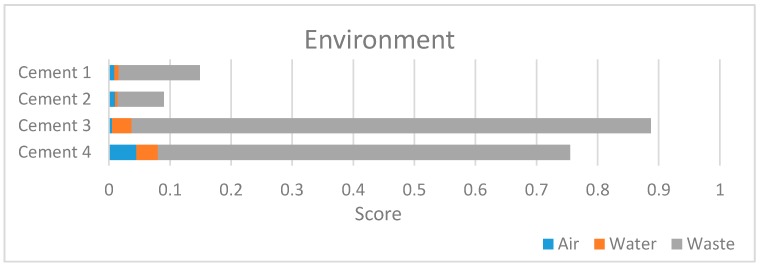
Level II: environmental results.

The same procedure should be adopted for other environmental criteria of level IV. Figure 3 shows the results.

Figure 3 highlights the waste production has a relevant role in environmental impact in all examined cements. Waste management is more efficient in plants which produce cement 3 and 4. The main waste products are paper and cardboard packaging, wooden and plastic packaging, mineral oils, electrical equipment and refractory materials out of order. In order to reduce their impact on the environment and allow their recovery, it is necessary to provide an advanced waste collection. In the Italian cement sector this practice covers 36% of total solid waste production [41].

Atmosphere emissions are related to implementation of Best Available Techniques (BAT) for cement industry, as reported in the European Reference document for the “Production of Cement, Lime and Magnesium Oxide” [42]. Particularly, the use of secondary fuels or raw materials (e.g., waste or by-products of mechanical and chemical industries) allows an integrated waste management approach, which saves natural and non-renewable resources and permits to recover waste in highly controlled conditions [43]. At this purpose, plant 4 has the best performance in terms of atmospheric emissions as consequence of:

- Applying BAT (dry process kiln with multistage preheating and precalcination—in plant 4-, instead of Lepol kilns which causes higher values of emissions and fuel consumption—in plants 1 to 3);
- High percentages of caloric substitution by means of alternative fuels (average value 16.3% for plant 4, on average 8.2% for plants 1 to 3).

As regard as the water environment, better performances of plant 4 respect to plants 1 to 3 are related to the environmental policies adopted by the company 4: it has practically zeroed the release of industrial water into the environment replacing open circuit system with closed loop systems.

**Figure 4:**
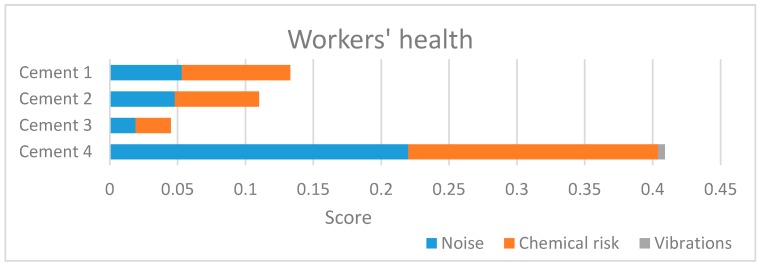
Level II: workers’ health results.

Figure 4 represents the results obtained about the workers’ health.

The influence of mechanical vibrations (transmitted both to the whole body and to hand/arm) on workers’ health has very low importance. The result is justified by the machines park of each company, which belongs to the latest generation [42].

**Figure 5:**
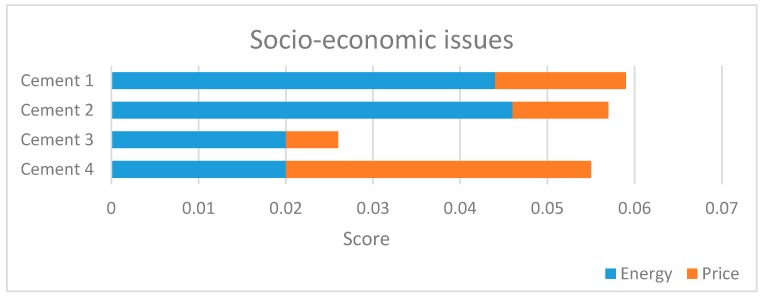
Level II: socio-economic results.

The chemical risk assumes an important role, as expected, since the operators come into contact with substances which significantly affect their health. The purchase of new equipment and the use of additives and chemicals not harmful to human health rewarded company 4. It is therefore confirmed the need for adopting protective and preventive measures to reduce chemical risks, especially for plant 3, which has the worst workers’ health performances. According to Figure 4, the noise in cement plants represents the second factor of risk after the chemical one: mechanical equipment produces high noise, especially if they are not compliant with noise regulations [44]. It is therefore important to quieter equipment and machinery, and use hearing protection to reduce this risk [45,46]. Figure 5 represents the results obtained about the socio-economic issues.

As regard as the energy consumption, the analysis considered water and electricity consumption. Most of the observed differences are related to water consumption, since electricity consumption for cements which have the same *R_ck_* is not appreciable [47]. The realization of closed rings circuits permits to substantially reduce water consumption, during the cooling phase [48]. Plant 2 has the best results, also related to the reuse of rainwater in the production cycle, while companies 3 and 4 have the worst performances because they did not implement measures to recycle and reduce water consumption.

As regard as the unit price, cement 4 is the most convenient, the opposite of cement 3 which is the most expensive.

The results shown in Figures from 3 to 5 have been summed for each criterion listed in level I. This procedure allowed the selection of best cement, as represented in Table 25.

**Figure 6:**
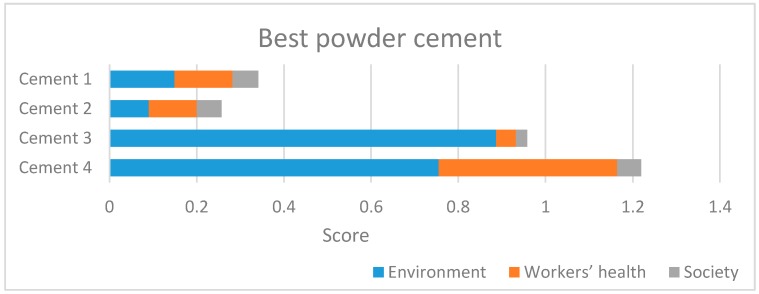
Level I: comparison between cement powders.

Figure 6 graphically represents the final results listed in Table 25. For each cement powder the contribution of each criterion of level I is represented.

Cement 4 is the most sustainable, and its performance is affected by the workers’ health criterion. Indeed, this contribution is 0.409, more than 33.4% of the overall result, and considerably more than the workers’ health contribution for the other cements (Figure 4). The results highlight the importance of considering more criteria in the analysis:

- Considering only environment (blue lines), cement 3 would be the best;
- Considering only socio-economic (green lines) results, cement 2 would be the best.

The AHP analysis ensured a good compromise between the three criteria (environment, workers’ health and society) applied on the cement production of four Italian companies, which are already placed at a high level in terms of technological and procedural innovation [49].

As it can be observed from all previous analyses, the results obtained from the AHP analysis could be useful to support the process of deciding the “best option”, because it allows a comprehensive, critical and unbiased analysis, which could be efficiently replicated at different level [50] in public bids.

## 4. Conclusions

Within the framework of efforts to find a compromise between economic growth and need to protect environment, the European Union has set targets for reducing greenhouse emissions, increasing energy efficiency, promoting use of renewable resources and reducing waste production. The environment is only one of the aspects to be considered in the decision phases, because the design choices involve multiple aspects ranging from legislative to technical-structural issues, from purely economic to human health criteria.

In this sense, the use of multi-criteria decision-making methods can overcome the limits of traditional decision-making approaches that lead one to overlook certain performances or not to give correct importance to others. These methods provide support to those who should make choices, requiring simple tasks in order to reach an objectively valid and unbiased choice.

This paper presents an application of the Analytic Hierarchy Process on four different cement powders produced by Italian companies. The method complies with objective of evaluating various options according to multiple and often conflicting features: it consists of pairwise comparisons between all considered parameters, which are sorted by non-dimensional values. The analysis consists of four levels: (1) the level I, which coincides with overall “goal” of the study: the best cement; (2) the level II, which considers environment, human health and socio-economic aspects; (3) the level III, which includes eight sub-criteria of level 2; (4) the level IV, which consists of 15 numerical sub-criteria of level 3.

The presented analysis allows comparing the overall sustainability of 1 Mg of 32.5 grade cement produced at four different plants. The AHP study demonstrates that considering only environmental issues, cement 3 would be the best; considering only socio-economical results, cement 2 would be the best, while assessing the whole sustainability of each cement, cement 4 is the best. This shows that it is incorrect to consider only one indicator to select the best material among several alternatives, mostly when workers’ health is considered. This result is paramount because the growing attention for sustainable construction issues needs for a comprehensive, simple and versatile methodology to be applied at different level in public bids.

## Figures and Tables

**Table 1:** Impact categories examined in the study.

Impact Categories	Unit of Measure	Cement 1	Cement 2	Cement 3	Cement 4
Global Warming Potential, *GWP*	kg CO_2_ eq.	737.59	664.6	785	611
Ozone Depletion Potential, *ODP*	kg CFC-11 eq	3.39 × 10^−5^	3.41 × 10^−5^	5.16 × 10^−5^	1.00 × 10^−5^
Photochemical Ozone Creation Potential, *POCP*	kg C_2_H_4_ eq	0.12	0.11	0.26	0.07
Eutrophication Potential, *EP*	kg PO_4_ eq	0.45	0.43	0.28	0.18
Non-hazardous Waste, *nHW*	kg	158	188	0.053	6.11
Hazardous Waste, *HW*	kg	0.12	0.14	0.1	0.0307
Renewable resources with energy content, *RRE*	MJ	199	192.77	275	256
Non-renewable resources with energy content, *nRRE*	MJ	7185	6799.01	6525	3080
Electricity, *E*	kWh	189.41	183.17	132	175
Water consumption, *W*	m^3^	0.481	0.573	0.705	0.674

**Table 2:** Human health and economic data examined in the study.

Parameter	Unit of Measure	Cement 1	Cement 2	Cement 3	Cement 4
Exposure to Cr(VI)	-	Not relevant	Not relevant	Low	Not relevant
Exposure to FCS	-	Not relevant	Not relevant	Low	Not relevant
Exposure to allergising substances	-	Low	Low	Average	Not relevant
Noise	dB (A)	80	80.5	82	79
WBV	m/s^2^	0.5	0.5	0.65	0.4
HAV	m/s^2^	1	1	1.2	1.05
Unit price	€/m^3^	136	148	150	112

FCS: free crystalline silica; WBV: whole body vibrations, HAV: hand-arm vibrations.

**Table 3:** Scale of importance by Saaty [36].

Intensity of Importance	Definition of Importance	Condition
**1**	Equal	Both elements contribute equally to the goal
**3**	Moderate	Experience and judgement moderately favour one of the elements
**5**	Strong	Experience and judgement strongly favour one element over another
**7**	Very strong	Judgement very strongly favour one element over another
**9**	Extreme	One element is favoured very strongly over another, as confirmed by evidence
**2,4,6,8**	Intermediate values between odd adjacent values	Compromise is necessary

**Table 4:** Example of matrix *C*.

	*O*_1_	*O*_2_	*O*_3_	*O*_4_
*O*_1_	*1*	*m*_12_	*m*_13_	*m*_14_
*O*_2_	*1/m*_12_	*1*	*m*_23_	*m*_24_
*O*_3_	*1/m*_13_	*1/m*_23_	*1*	*m*_34_
*O*_4_	*1/m*_14_	*1/m*_24_	*m*_34_	*1*

**Table 5:** Pairwise comparison matrix for level II (C,_II_).

Level II	Environment	Workers’ Health	Society
**Environment**	1	0.5	2
**Workers’ health**	2	1	2
**Society**	0.5	0.5	1

**Table 6:** Normalized pairwise comparison matrix for level II (N,_II_).

Level II	Environment	Workers’ Health	Society
**Environment**	0.29	0.25	0.40
**Workers’ health**	0.57	0.50	0.40
**Society**	0.14	0.25	0.20

**Table 7:** Weight vector for level II (W_,II_).

Level II	Weights
**Environment**	0.312
**Workers’ Health**	0.490
**Society**	0.198

**Table 8:** Random Consistency Index [36].

*RCI*	0	0	0.58	0.9	1.12	1.24	1.32	1.41	1.45	1.49
***n***	1	2	3	4	5	6	7	8	9	10

*RCI*: random consistency index.

**Table 9:** Consistency analysis for level II.

***CI***	0.03
***RCI***	0.58
***RC***	0.05

*CI*: mean consistency index; *RC*: ratio consistency.

**Table 10:** Pairwise comparison matrix for level III, environment analysis (C,_IIIe_).

Level III, Environment	Atmosphere	Waste	Water Environment
**Atmosphere**	1	0.33	1
**Waste**	3	1	3
**Water environment**	1	0.33	1

**Table 11:** Normalized pairwise comparison matrix for level III, environment analysis.

Level III, Environment	Atmosphere	Waste	Water Environment
**Atmosphere**	0.20	0.20	0.20
**Waste**	0.60	0.60	0.60
**Water environment**	0.20	0.20	0.20

**Table 12:** Weight vector for level III (W_,IIIe_).

Level III, Environment	Weights
**Atmosphere**	0.200
**Waste**	0.600
**Water environment**	0.200

**Table 13:** Consistency analysis for level III, environment analysis.

***CI***	0
***RCI***	0.58
***RC***	0

**Table 14:** Pairwise comparison matrix for level III, Workers’ health analysis (C,_IIIW_).

Level III, Workers’ Health	Chemical Risk	Noise	Mechanical Vibrations
**Chemical risk**	1	5	6
**Noise**	0.2	1	3
**Mechanical vibrations**	0.17	0.33	1

**Table 15:** Weight vector for level III (W,_IIIw_).

Level III, Workers’ Health	Weights
**Chemical risk**	1
**Noise**	0.2
**Mechanical vibrations**	0.1

**Table 16:** Pairwise comparison matrix for level IV, atmosphere analysis (C,_IVa_).

Level IV, Atmosphere	*GWP*	*ODP*	*POCP*
***GWP***	1	0.5	2
***ODP***	1	1	0.5
***POCP***	0.5	2	1

**Table 17:** Normalized pairwise comparison matrix for level IV, atmosphere analysis.

Level IV, Atmosphere	*GWP*	*ODP*	*POCP*
***GWP***	0.40	0.14	0.57
***ODP***	0.40	0.29	0.14
***POCP***	0.20	0.57	0.29

**Table 18:** Weight vector for level IV (W_,IVa_).

Level IV, Atmosphere	Weights
***GWP***	0.371
***ODP***	0.276
***POCP***	0.352

**Table 19:** Consistency analysis for level III, environment analysis.

***CI***	0.06
***RCI***	0.58
***RC***	0.09

**Table 20:** Pairwise comparison matrix for environmental sub-criteria *GWP*.

*GWP*	Cement 1	Cement 2	Cement 3	Cement 4
**Cement 1**	1	0.33	3	0.20
**Cement 2**	3	1	5	0.33
**Cement 3**	0.33	0.20	1	0.17
**Cement 4**	5	3	6	1

**Table 21:** Normalized pairwise comparison matrix for environmental sub-criteria *GWP*.

*GWP*	Cement 1	Cement 2	Cement 3	Cement 4
**Cement 1**	0.11	0.07	0.20	0.12
**Cement 2**	0.32	0.22	0.33	0.20
**Cement 3**	0.04	0.04	0.07	0.10
**Cement 4**	0.54	0.66	0.40	0.59

**Table 22:** Weight vector of *GWP.*

*GWP*	Weights
**Cement 1**	0.125
**Cement 2**	0.268
**Cement 3**	0.061
**Cement 4**	0.546

**Table 23:** Consistency analysis for *GWP.*

***CI***	0.05
***RCI***	0.90
***RC***	0.08

**Table 24:** Sorting matrix of alternatives, atmosphere.

Level IV	*GWP*	*ODP*	*POCP*
**Cement 1**	0.125	0.138	0.142
**Cement 2**	0.268	0.138	0.142
**Cement 3**	0.061	0.050	0.062
**Cement 4**	0.546	0.673	0.654

**Table 25:** Contribution of elements of level II to the main goal.

Best Cement	Environment	Workers’ Health	Society	Final Results
**Cement 1**	0.149	0.133	0.059	**0.341**
**Cement 2**	0.090	0.110	0.057	**0.257**
**Cement 3**	0.887	0.045	0.026	**0.958**
**Cement 4**	0.755	0.409	0.055	**1.220**
